# Barriers to, and facilitators of, the adoption of a sugar sweetened beverage tax to prevent non-communicable diseases in Uganda: a policy landscape analysis

**DOI:** 10.1080/16549716.2021.1892307

**Published:** 2021-04-20

**Authors:** Gemma Ahaibwe, Safura Abdool Karim, Anne-Marie Thow, Agnes Erzse, Karen Hofman

**Affiliations:** a Economic Policy Research Centre (EPRC), Makerere University, Makerere, Uganda; b SAMRC Centre for Health Economic and Decision Science - Priority Cost Effective Lessons for Systems Strengthening (PRICELESS SA), University of the Witwatersrand, School of Public Health, Johannesburg, South Africa; c Menzies Centre for Health Policy, School of Public Health, The University of Sydney, Sydney, Australia

**Keywords:** Fiscal policies, non-communicable diseases, sugar-sweetened beverage tax, policy analysis

## Abstract

**Background:**

Uganda is experiencing an increase in nutrition-related non-communicable diseases. Risk factors include overconsumption of sugar-sweetened beverages. Fiscal and taxation policies aim to make the consumption of healthier foods easier. However, the adoption and implementation of fiscal policies by countries are constrained by political and economic challenges.

**Objective:**

We investigated the policy and political landscape related to the prevention of nutrition-related non-communicable diseases in Uganda to identify barriers to and facilitators of the adoption of sugar-sweetened beverage taxation in Uganda.

**Methods:**

A desk-based policy analysis of policies related to nutrition-related non-communicable diseases and sugar-sweetened beverage taxation was conducted. Four key informant consultations (n = 4) were conducted to verify the policy review and to gain further insight into the policy and stakeholder contexts. Analysis was framed by Kingdon’s theory of agenda setting and policy change.

**Results:**

Nutrition-related non-communicable diseases were recognised as an emerging problem in Uganda. The Government has adopted a comprehensive approach to improve diets, but implementation is slow. There is limited recognition of the consumption of sugar and sugar-sweetened beverages as a contributor to the nutrition-related non-communicable disease burden in policy documents. Existing taxes on soft drinks are lower than the World Health Organization’s recommended rate of 20% and do not target sugar content. The soft drink industry has been influential in framing the taxation debate, and the Ministry of Finance previously reduced taxation of sugar-sweetened beverages. Maintaining competitiveness in a regional market is an important business strategy. However, the Ministry of Health and other public health actors in civil society have been successful (albeit marginally) in countering reductions in taxation, which are supported by industry.

**Conclusions:**

An established platform for sugar-sweetened beverage taxation advocacy exists in Uganda. Compelling local research that explicitly links soft drink taxes to health goals is essential to advance sugar-sweetened beverage taxation.

## Background

In Uganda, non-communicable diseases (NCDs) are an emerging challenge, accounting for 11–12% of the disease burden [1] and 33% of all deaths [2]. Globally, changes in diet and lifestyle have led to an increase in the prevalence of NCDs, such as obesity, diabetes, cardiovascular disease, and hypertension [3]. The food system in Uganda is in the early stages of transition, moving from rural, informal, and small-scale sellers/providers, to increased urbanization where regional supermarkets and fast-food chains are increasingly responsible for food sales [4]. Emerging evidence links this food transition to higher rates of certain nutrition-related NCDs (NR-NCDs). In 2014, the prevalence rates of hypertension and diabetes among the adult population (18–69 years) were reported to be 24% and 3.4%, respectively [5]. In addition, 7.2% of women were obese in 2016, almost triple the prevalence of 2.7% in 2001 [6]. While the prevalence of NR-NCDs in Uganda is lower than that in other sub-Saharan Africa countries, health facilities are challenged by the burden of disease [7,8]. For example, a recent study of the capacity of public sector health care facilities showed that none of the 53 facilities assessed had access to all of the essential medical devices recommended by the World Health Organization (WHO) Package of Essential Non-communicable Disease Interventions for Primary Health Care in Low-Resource Settings [8].

The increase in consumption of sugar-sweetened beverages (SSBs) has been identified as a major contributor to the rise of, amongst other NCDs, obesity and type-2 diabetes [9]. SSBs contribute a significant proportion of the daily energy consumption per person [10]. There is growing evidence of the increasing availability and consumption of SSBs in Uganda [11]. From 2011 to 2012, the soft drink market in Uganda changed substantially. Three new companies entered a market that had previously been dominated by Century Bottling Company, which bottled Coca-Cola and Crown Beverages [12]. The soft drink and beverage market has continued to grow in Uganda [13]. The value of carbonated soft drink sales – the largest and fastest growing category of non-alcoholic drinks – grew at a rate of 12.4% during the 2015–2018 period, and is projected to grow at an annual average rate of 10.9% from 2019 to 2022 [13]. This is attributable, in part, to relatively cheap marketing (in comparison with industrialised environments), concerted advertising, and with little regulation.

It is therefore clear that regulatory actions to protect public health and prevent the costly burden of NR-NCDs need to be taken [14]. A proposed cost-effective regulatory measure is the introduction of SSB taxation to reduce the consumption of sugar. SSB taxation has been shown to be an effective mechanism to reduce SSB (and sugar) consumption and encourage the consumption of healthier alternatives, such as water [15,16]. In lower-income countries, where people tend to spend a larger proportion of their income on food, demand for some, usually non-staple, foods and beverages can be very price elastic [17]. However, significant political and economic challenges are associated with the adoption and implementation of fiscal policies. For example, South Africa’s SSB tax was announced in 2016 but only came into effect in 2018 and at a lower rate than initially proposed – mostly due to industry push back [18,19].

To understand how existing fiscal instruments can be leveraged to address public health concerns, it is important to understand the context in which policies for prevention of NR-NCDs are developed and implemented. The aim of this study was to understand the political and evidence landscapes in which policies for prevention of NR-NCDs are being developed in Uganda, and to identify contextual factors within the policy landscape that might influence the adoption of SSB taxation policies.

## Methods

We conducted a prospective desktop policy analysis [17] as part of a broader regional study which, analysed the barriers to, and facilitators of, the adoption of SSB tax in seven sub-Saharan African countries [20]. The detailed methodology used to conduct this analysis is outlined in the study design paper [20]. Nine existing government policies in Uganda, relevant to SSB taxation and the reduction of NR-NCD in the general population, were reviewed. These policies were drawn from government sectors, including economic, fiscal, health, agriculture, and others.

The lead author conducted consultations with four key stakeholders to 1) verify that we had identified all relevant policy documents, 2) ensure that we had correctly interpreted the policy content, and 3) gain additional understanding of the policy and political context. Consultations were strictly focused on publicly available information that was part of the respondents’ normal work and responsibilities. Consultations were conducted with public health actors within government (n = 2), the government economic sector (n = 1), and a consumer interest community service organisation (n = 1).

Data were organised into pre-determined data matrices [20]. Results were then analysed in three categories, using Kingdon’s multiple streams of policy change theory [21].

## Results

The Kingdon framework [21] was used to analyse the data related to NR-NCDs and SSBs and to frame our results. The categories included: evidence of the problem of NR-NCDs and SSB taxation (category 1), the existing policy (category 2), and the political context related to the key stakeholders (category 3) [21].

### Category 1: Evidence of the problem formulation of NR-NCDs and SSBs in Uganda

Data are important for monitoring trends of NR-NCDs and the effects of policy interventions. There were several sources of NR-NCD prevalence data; however, these data were not directly comparable as they were collected using different methods. The 2014 STEP-wise approach to surveillance (STEPS) survey provided baseline data on the prevalence of hypertension, diabetes, and cardiovascular disease, and cholesterol and high-density lipoprotein (HDL) cholesterol [5]. The Ugandan national household surveys that are routinely conducted every three to 4 years included the collection of data on self-reported diabetes, hypertension, and heart disease. In addition, international sources, such as the World Bank Development Indicators (WDI) database and WHO NCD country profile reports were good sources of data for the NR-NCD disease burden [22]. The inconsistencies between data sets may pose challenges to monitoring and evaluation efforts by the Government where interventions aimed at reducing the burden of NR-NCDs are implemented.

Data about diet and anthropometry were extracted from national household surveys conducted by the Uganda Bureau of Statistics. These included extensive data on household food expenditure, sources of food, prices of food, fruit and vegetable consumption, diet diversity, and food security status [23]. Data were also collected on the consumption of sugary beverages, particularly soda and juices (both fresh and packed), tobacco, alcoholic drinks, and diet-related risk factors, such as salt and sugar consumption [23]. In addition, the Uganda Demographic and Health Survey (DHS) contains information on anthropometrics and micronutrient deficiencies [23]. An advantage of these datasets is that they include many rounds of data, dating as far back as the 1990s.

With regard to industry data, a number of sources exist but access is not free; examples include Euromonitor and Fitch Solutions. This presents challenges in understanding the role that the SSB and sugar industries play in the local economy. As a consequence, there is limited local evidence that explicitly quantifies the contribution of specific risk factors, such as SSBs, to the local NR-NCD disease burden, and limited data about the value of introducing a public health tax on SSBs and the likely impact on consumption, employment and, eventually, NR-NCDs.

### Category 2: Policies on NR-NCDs and SSBs

Table 1 summarises the existing policies in relation to NR-NCDs in Uganda. The overarching national planning and health sector policies of Government acknowledge that NCDs, including NR-NCDs, are increasingly becoming a problem in Uganda and lead to high death rates, disabilities, and associated high medical costs.

*“Non-communicable diseases such as high blood pressure, cancers, diabetes, injuries and disabilities, genetic diseases and others are on the increase”* [24, *p.32]*

Uganda is experiencing an upsurge of non-communicable diseases; these diseases lead to high death rates, various disabilities and high medical costs (examples mentioned are diabetes, hypertension, obesity, cardiovascular diseases, some cancers). [25, p.22]

**Table 1.** Reviewed policies and lead agencies related to NR-NCDs and SSB taxation in Uganda

Policy document	Content in relation to nutrition and/or NCDs	Responsible for action (lead agency)
**Whole of Government**		
Vision 2040 [39]	There should be a *“Paradigm shift from curative to preventive health system; the main thrust of this paradigm is an empowerment of households and communities to take greater control of their health by promoting healthy practices and lifestyles”*	National Planning Authority and all ministries, departments, agencies and local governments
Second National Development Plan (2015/16–2019/2020) [24]	*“Promote healthy lifestyles that contribute to prevention or delay of occurrence of NCDs”*	National Planning Authority and all ministries, departments, agencies and local governments
**Economic and fiscal related policies**		
Excise duty amendment Acts (2018/19) [40]	Excise tax on non-alcoholic beverages (excluding fruit and vegetable juices) 12% or UGX 200 (approx. 0.05 USD) per litre, whichever is higher Fruit and vegetable juice (except juice made from at least 30% of pulp from fruit and vegetables grown in Uganda) 13% or UGX 300 (approx. 0.08 USD) per litre, whichever is higher	Ministry of Finance, Planning and Economic Development Uganda Revenue Authority
**Cross-sectoral policies**		
Uganda Food and Nutrition Policy (2003) [25]	*“Promote the nutritional status of the people of Uganda through multi-sectoral and coordinated interventions that focus on food security, improved nutrition and increased incomes”*	Ministry of Health, Ministry of Agriculture, Animal Industry and Fisheries, office of the prime minister
**Health Sector Policies**		
National Health Sector Development Plan (2014/15–2019/20) [26]	One of the proposed interventions is the provision of NCD prevention and control services. To create community awareness of the right foods to eat for good nutrition and to promote their production	Ministry of Health
Second National Health Policy (2010) [41]	*“Improve people’s awareness about health and related issues in order to bring about desired changes in knowledge, attitudes, practices and behaviours regarding the prevention and control of major health and nutrition problems in Uganda”*	Ministry of Health
**Agricultural Sector Policies**		
National Agricultural Policy (2013) [42]	One objective is to promote the production of nutritious foods, including indigenous foods, to meet household needs and for sale and consumption of nutritious foods	Ministry of Agriculture, Animal industries and fisheries
Agriculture sector strategy plan (2015–2020) [43]	“*Improve food and nutrition security by enhancing consumption of diverse diets at household level”* (page 65). Also fruit and vegetables are among the Plan’s 12 strategic and priority commodities	Ministry of Agriculture, Animal industries and fisheries
Uganda Food and Nutrition Strategy (2005) [25]	*“Monitor trends of NCDS and promote healthy diets and healthy lifestyles”*	Ministry of Agriculture, Animal industries and fisheries Ministry of Health

Nutrition policies, rather than development and health policies more broadly, explicitly linked nutrition and NCDs. The National Development Plan attributed NCDs to genetics and lifestyle changes, without specifying risk factors such as sugar, salt, and fat consumption [24]. Similarly, the Second National Health Policy (2010) attributed NCDs to multiple factors, such as adoption of unhealthy lifestyles, increasing life expectancy, and metabolic side effects resulting from lifelong antiretroviral treatment. Nutrition-related policies attributed the increased prevalence of NCDs to changes in diets. For instance, the Uganda Food and Nutrition Strategy of 2005 attributed the increased rate of NCDs to changes in dietary habits, over-consumption of energy-rich foods, smoking, recreational drug use, and increasingly sedentary lifestyles.

There was limited recognition of sugar and SSB consumption as a notable contributor to NR-NCDs. Most of the NR-NCD-related policies (at government and sectoral level – health, agriculture) used the general term ‘unhealthy diets’ without elaborating on what this covered. However, the National Health Sector Development Plan (2014/15–2019/20) (HSDP) and the Department of NCDs specifically identified sugar as a concern, *‘the rapid spread of risk factors, such as tobacco use and physical inactivity, unhealthy diets with lots of sugars, fats and salt, and alcohol abuse, along with ageing populations and unplanned urbanization, have a profound influence on health and wellbeing globally’ [26*, p.21]. Similarly, the Ministry of Health’s Community Health Department website states, *‘eating healthy foods with less fat, less sugar and less salt’* as one remedy for NR-NCDs [27].

In general, it can be seen that Uganda is taking a multi-sectoral approach to NCDs by integrating NR-NCD prevention policy into various sectoral policies: *‘tackling the burden of NCDs will require action in multiple sectors’* [26, p.21]. Although this is not co-ordinated through a dedicated or explicit policy or strategy aimed at controlling NR-NCDs, there are already supportive policies in the health, nutrition, agricultural and fiscal sectors (Table 1). One solution that was common across most policy documents was the proposal to use social marketing and health promotion campaigns to create awareness at the community level on foods to eat for good nutrition status and to promote the production of healthy foods.

An intention to use fiscal policies as a way of reducing the burden of NR-NCDs was evident. The most comprehensive proposal was detailed in the now dated Uganda National Food and Nutrition Strategy of 2005. It proposed the implementation of both supply- and demand-side policies to reduce obesity in Uganda.

“Where judged effective, implement both supply-side and demand-side policies to slow increases in obesity in Uganda. Supply-side policies might include market interventions to encourage the production of healthier foods, particularly fruits and vegetables, and controls on the fat content of processed foods, for example, while demand-side policies include changing the relative prices of healthy and unhealthy foods, providing information on healthy diets, and promoting healthy, active lifestyles” [25,25,p.28].

However, implementation is lacklustre. Our review and consultations did not identify examples of market or other interventions that have been adopted in respect of NCDs, despite the strategy having been in place for more than a decade. Given the recognition of NCDs in the policy documents and several proposals on how to improve the consumption of healthy foods, there was no clear evidence of strong leadership by Government in terms of implementation.

Uganda has adopted an excise duty tax, ranging from 12% to 15%, depending on the type of beverage (Table 1) but SSBs and non-SSBs are not distinguished. The highest rate (15%) is charged on powder for reconstitution to make juice or dilute-to-taste drinks, excluding pulp. There is a tax exemption on fruit and vegetable juice made from at least 30% of pulp and fruit from Uganda. The exemption is aimed at promoting the use of local raw materials in the production of beverages. These taxes are premised on revenue generation and related economic reasons, as opposed to health concerns. For example, the reason behind the excise duty of 15% on all juices in 2018/19 was to widen the tax base and enhance revenue collection [28]. Ring-fencing of collected revenues is uncommon in Uganda, although 2% of the tax revenue from beers, spirits, waragi (a Ugandan homemade gin), soft drinks, and bottled water has been earmarked for HIV/AIDS prevention and treatment, provided for under the HIV/AIDS Trust Fund Act of 2014 [29].

### Category 3: The political context of key stakeholders

In line with our theoretical framework, our analysis of the political landscape focussed on actors and their efforts to influence policy. The reviewed policy documents and other related documentation identified the Ministry of Health (Department of Community Health), Ministry of Finance (Department of Tax policy), Parliament (Committee on Finance), and soft drink industry stakeholders as major actors for SSB taxation policy change in Uganda. As summarised in Table 2, Government and industry are the highly influential stakeholders, with civil society and academia having lower vested interests and influences on SSB taxation.

**Table 2.** Stakeholders, vested interests, and level of influence in relation to SSB taxation

Stakeholder Type	Example	Reason for interest in the SSB taxation	Level of influence – rationale
**Government**			
Cabinet	President and cabinet	Role of industry in the economy (jobs and GDP growth) and public health concerns	High (policy makers: executive arm of government)
Ministry of Health	Minister of health and state minister for health	Responsible for providing promotive, preventive and curative, services to the population	High (policy maker:member of executive arm of Government)
Ministry of Health	Department of NCDs	Their overriding mandate is prevention, detection and management of NCDs	High (policies originate from such technical units)
Ministry of Finance,	Commissioner for Tax Policy	Revenue generation to finance government budget	High (responsible for initiating, evolving and formulating tax policies)
Parliament	Committee on Finance	Role of industry in the economy (jobs and Gross Domestic Product (GDP) growth)	High (advises parliament on proposed tax measures)
**Civil society**	Uganda NCD Alliance, Tax Justice Alliance Uganda	Concerned about the increased prevalence of NCDs and interested in proposals aimed at reducing NCDs	Medium (can only advocate)
**Academia**	Universities and research institutes	Evidence generation	Medium (no direct role in policy making)
**Industry**	Coca-Cola, Pepsi cola and Riham	Profit maximization through low tax payments and high sales	High (role in the economy and strong lobbying power)

Previous levies on sugary products have not been retained. A tax introduced in the 2017 financial year on sugar confectionaries was repealed on the basis that it impacted the competitiveness of Uganda’s confectionaries in the East African region. In 2018, the Minister of Health objected to the proposal from the Finance Committee to cut the tax rate on non-alcoholic beverages (such as soda) from 13% to 10%. The tax was ultimately reduced by one percentage point, to 12%, due to industry pressure. The agreed plan of action is to gradually reduce the tax to 10%. The chief executive officer of Coca-Cola Uganda indicated how the economic interests of the soft drink industry underpinned this decision, stating:

*“This [USD 15 million] investment was made on a promise of reducing taxes (on soft drinks), from 13% excise duty to 12% in the 2018/19 financial year, and we are glad it was implemented. This is confirmation that a favorable tax regime can attract more investment for the industry.”* [30]

Table 3 summarises the strategies adopted by the soft drink industry to maintain its market and profitability. These strategies include information and messaging; advertising and financial incentives (such as sports and other sponsorships); and self-regulation and constituency-building through corporate social responsibility, to garner support and acceptance among policymakers and the population at large. The industry relies heavily on its economic importance related to investment, jobs, and revenue generation to negotiate favourable tax rates. The dominant narrative from industry players is that an SSB tax will create significant uncertainty for the industry, and will prevent or dampen prospects of further growth and investment.

“*We shall experience job cuts due to limited sales volumes. We shall also not be able to expand production and create more jobs. The government will also lose out on taxes in the long run.”* [31]

**Table 3.** Industry strategies to sustain the soft drinks market and profitability

Tactic	Findings	Evidence
Information and messaging	Industry lobbies directly to influence legislation so that it is favourable to the industry	**Beverage firms decry high taxes: b**y Nassali Fatiah, 22 February 2018 [44] **Producers of soft drinks protest tax increments on products: b**y Jonathan Adengo, 26 May 2017 [31] **President Museveni meets executives of Soft drinks companies**: by Linda Nabusayi, 13 April 2016 [45] **Against excise duty on carbonated soft drinks**: Parliament Watch, 27 ^h^ November 2014 [46]
	Industry stresses its importance in terms of jobs and revenue generation	“We employ about 1,800 Ugandans in our three plants and support more than 90,000 businesses across our extensive retail distribution network. Coca-Cola Beverages Africa is proud to make these contributions on top of paying taxes to the tune of more than Uganda Shillings 140 billion (USD 38 million) annually. We are serious about doing business in Uganda and supporting this economy.” [47] “We shall experience job cuts due to limited sales volumes. We shall also not be able to expand production and create more jobs. The government will also lose out on taxes in the long run.” [31]
Constituency building	Industry uses corporate social responsibility for constituency building	**Here is Coca-Cola’s sponsorship package to the MTN marathon**: by Hassan Kibirige, 17 November 2018 “Coca-Cola is always proud to be part of a good cause and we are passionate about improving maternal health – this year’s marathon theme, both as a Company and at a personal level. This relationship shall continue for many years to come”. [48]
Policy substitution	The industry is trying to self regulate itself through introduction of zero sugar products	**Coke unveils more sugar-free soda: b**y Isaac Khisa, “The major driver (for product diversification) is the changing consumer habits, tastes and preferences … (and) the increased consciousness of health – and that is why we present more healthy options for the more health-conscious consumer to choose from when they need refreshment.*”* [49]

Source: Authors’ compilation based on the approach in Mialon et al., (2015) [50]

Government stakeholders do not agree about the benefits of SSB taxation and this has inevitably made the lobbying of the soft drink industry much easier. The Ministry of Finance acts to protect Ugandan business and its regional competitiveness.

*“The excise tax rate on soft drinks should be reduced from 13% to 12% to increase Uganda’s competitiveness, protect and grow the local soft drink industry”* [32].

Given the development challenges that Uganda faces (poverty and youth unemployment) [6], the economic imperatives override health concerns. The rationale for the introduction of the SSB tax was not health-related but, rather, economic. The justification for the reduction in excise tax for non-alcoholic beverages in 2018/19 was to enhance Uganda’s competitiveness and protect the industry from unfair competition from other East African countries with an average tax of 10% [33]. It was argued that having a higher tax in Uganda would reduce the industry’s competitiveness because there would be smuggling of cheaper beverages from neighbouring countries in east Africa [33].

Civil society organisations (CSOs) in Uganda have also taken an interest in SSB taxation. There are two civil society umbrella organisations. The first is the Uganda Non-Communicable Disease Alliance, which brings together the Uganda Cancer Association, the Uganda Diabetes Association, and the Uganda Heart Foundation. Its mandate is to raise awareness about the prevention, detection, and management of NCDs in the general population. The second organisation includes the Tax Justice Alliance Uganda (TJAU), SEATINI, and Action Aid, which objected to the Government’s proposal to reduce the drink taxation from 12% to 11% for the financial year (2019/20). The TJAU presented an argument to Parliaments’ Committee on Finance, that most non-alcoholic beverages are luxury goods with negative effects on the health of Ugandans. However, the tax rate was not reduced to 11%, indicating the success of CSO advocacy and lobbying.

*“Non-alcoholic beverages such as soda, energy drinks and non-fruit juices are goods, which with increased consumption could negatively affect the health of the consumer”* [34].

## Discussion

We sought to understand the potential opportunities for, and challenges to, increasing existing SSB taxation and improving NR-NCD-related policies in Uganda. From the document review, we found that the Government has recognised the need for a comprehensive approach to address NR-NCDs, but implementation is slow. Although there are taxation policies that could be leveraged upon to tax SSBs, they are currently used for revenue-generation rather than health purposes, and the taxation rate is well below the 20% recommended by the WHO [15]. The taxation rates have also been revised several times and have been adjusted downwards by the revenue services to maintain the competitiveness of the Ugandan drink industry in the region.

The soft drink industry has been an active stakeholder in relation to SSB policies and has successfully influenced fiscal policies in the Ministry of Finance. However, policymakers from the Department of Health and CSOs have had some success in countering changes to fiscal policies to dis-incentivise consumption of unhealthy commodities. Although the existing tax on beverages is aimed at revenue-generation rather than reducing NR-NCDs, it is, nevertheless, a platform that could potentially be leveraged to introduce a taxation policy that could significantly reduce consumption of SSBs. A 20% tax on sugary beverages has been associated with significant reductions in the burden of obesity in South Africa [35], with the potential for significant reductions in mortality [36,37]. If existing taxation policies were aligned to the recommendations of a higher tax rate and levied only on sugary beverages, these reductions could be realised in Uganda.

The potential benefits and any potentially unfavourable outcomes of a higher SSB tax in Uganda are not well researched or understood. Although evidence from other low – and middle-income countries can provide guidance on the potential impact of increasing SSB tax, local evidence is likely to be instrumental in the adoption of a policy. Consequently, local research and analysis about the impacts of progressively increasing the tax of SSBs are necessary. This could include modelling studies on the likely impacts on consumption, employment, and, eventually, NR-NCDs.

In other contexts, linking such taxes explicitly to health goals has been shown to improve public support [17]. To strengthen Ugandan SSB fiscal policies, empirical evidence that links SSB taxes to health goals is necessary to allay concerns about the negative impacts of taxes on economic variables, such as employment and the gross domestic product (GDP). Collection and analysis of local evidence on NR-NCDs and SSB taxation may also improve political buy-in. Preparing this case is a critical role for public health civil society actors and is necessary to counteract pressure from well-resourced and well-positioned industry players.

Given the lobbying power and industry tactics documented in this paper, any further increase in the existing tax is likely to be met with strong resistance from industry players. Currently, the lobbying of industry is enabled by the incoherence between the objectives of public health and economic-related policies. The strong narrative provided by industry about its important role in the economy and the potential harm of taxation appeals to the primary concerns of the economic sector. Similar arguments were raised in opposition to the South African SSB tax [18]. However, these claims are often greatly exaggerated. SSB taxes are likely to generate a positive net increase in jobs, in spite of a small decrease in jobs in the beverage sector [15]. This is because consumers redirect their purchases to other untaxed or lower-axed goods, thus stimulating demand and, subsequently, growth in other non-beverage sectors [15]. The soft drink industry is a regionalised business that is sensitive to taxation precedents in other countries. Public health actors should consider the opportunity to act regionally, as well as locally, with respect to SSB taxation. For example, there is a policy in Uganda that earmarks 2% of the total revenue from beers, spirits, *waragi*, soft drinks, and bottled water for the HIV/AIDS Trust Fund [29]. Opportunities exist to set regional precedents for taxation policies applicable to the food and beverage industry. Other options that may be considered relate to subsidies for healthier foods, particularly fruit, and vegetables. Evidence shows that subsidies for fresh fruit and vegetables that reduce prices by 10–30% are effective in increasing fruit and vegetable consumption [15].

### Limitations

As this study was limited to a desk-based review, it had a number of limitations. Our reliance on documents in the public domain limited the kind of information we were able to obtain. However, through consultations, we ensured that the reviewed documents were considered by key stakeholders to be complete, up-to-date, and correctly interpreted. We also drew on media reports to provide data on political activity, which has not previously been done in Uganda. Nevertheless, we encountered substantial difficulties in obtaining information about private actors, specifically SSB industry actors, that were publicly available. To overcome this limitation, we purchased a dataset on the beverage industry in Uganda. However, even with this additional information, we found limited information about the political context surrounding SSB taxation. Future research could include primary data collection to better understand stakeholder views and the political environment.

## Conclusion

The findings from Uganda are not unique; most countries that have tried to implement SSB taxes have faced considerable political and industry opposition. In South Africa, industry opposed the tax on the basis of employment and other economic impacts [38]. The soft drink industry is important to Uganda’s economy, and government actions prioritise market competitiveness. The Ministry of Health and CSOs have track records for challenging reductions in the current levels of soft drink taxation in the interest of public health and the reduction of NR-NCDs. The generation of a compelling Ugandan case for SSB taxation and NR-NCD reduction, using local data, will strengthen the advocacy case. A proactive approach towards policy coherence between government health and economic ministries will also weaken the lobbying position of industry.

## Paper context

Uganda is experiencing an increase in nutrition-related non-communicable diseases and taxation of sugary beverages can prevent this. Private actors have influenced taxation policies related to sugary beverages. Although the policy environment is supportive of the tax, the lack of information related to both risk factors and private actors presents a barrier to its adoption. Local research is needed to support the adoption of a sugary beverage tax.
